# Digital Therapeutic Alliance With Fully Automated Mental Health Smartphone Apps: A Narrative Review

**DOI:** 10.3389/fpsyt.2022.819623

**Published:** 2022-06-22

**Authors:** Fangziyun Tong, Reeva Lederman, Simon D'Alfonso, Katherine Berry, Sandra Bucci

**Affiliations:** ^1^School of Computing and Information Systems, University of Melbourne, Parkville, VIC, Australia; ^2^Division of Psychology and Mental Health, School of Health Sciences, Manchester Academic Health Sciences Centre, University of Manchester, Manchester, United Kingdom; ^3^Complex Trauma and Resilience Research Unit, Greater Manchester Mental Health NHS Foundation Trust, Manchester, United Kingdom

**Keywords:** digital mental health, digital therapeutic alliance, mHealth, smartphone app, human-computer interaction

## Abstract

Fully automated mental health smartphone apps show strong promise in increasing access to psychological support. Therefore, it is crucial to understand how to make these apps effective. The therapeutic alliance (TA), or the relationship between healthcare professionals and clients, is considered fundamental to successful treatment outcomes in face-to-face therapy. Thus, understanding the TA in the context of fully automated apps would bring us insights into building effective smartphone apps which engage users. However, the concept of a digital therapeutic alliance (DTA) in the context of fully automated mental health smartphone apps is nascent and under-researched, and only a handful of studies have been published in this area. In particular, no published review paper examined the DTA in the context of fully automated apps. The objective of this review was to integrate the extant literature to identify research gaps and future directions in the investigation of DTA in relation to fully automated mental health smartphone apps. Our findings suggest that the DTA in relation to fully automated smartphone apps needs to be conceptualized differently to traditional face-to-face TA. First, the role of bond in the context of fully automated apps is unclear. Second, human components of face-to-face TA, such as empathy, are hard to achieve in the digital context. Third, some users may perceive apps as more non-judgmental and flexible, which may further influence DTA formation. Subdisciplines of computer science, such as affective computing and positive computing, and some human-computer interaction (HCI) theories, such as those of persuasive technology and human-app attachment, can potentially help to foster a sense of empathy, build tasks and goals and develop bond or an attachment between users and apps, which may further contribute to DTA formation in fully automated smartphone apps. Whilst the review produced a relatively limited quantity of literature, this reflects the novelty of the topic and the need for further research.

## Introduction

More than one in 10 people globally live with a mental health condition (1), and more than half of the population in middle- and high-income countries will experience mental health problems during their lives (2). Mental health problems cause high levels of distress and impair the quality of life for people experiencing them and their families (3, 4). In the UK, mental health problems cost about 14% of the total budget within the National Health Service (NHS) (5). Economists predict that by 2030, the global cost of treating common mental health problems, such as depression and anxiety, will scale up to US $147 billion (6).

Psychological interventions, such as cognitive and behavioral therapy (CBT), are effective in treating a range of mental health problems, in addition to or in place of pharmacological treatments (7). However, access to face-to-face psychological therapy remains low. According to the World Health Organization (WHO) (8), the number of mental health professionals trained to deliver therapy does not meet the level of need. In addition to inadequate staffing, various other barriers, such as stigma (9, 10), prevent people from seeking professional help for mental health problems.

Digital mental health interventions are considered viable solutions for increasing mental health service accessibility, decreasing government financial burden, and helping to overcome the barriers of stigma (11–13). Among the various types of digital health interventions, mental health smartphone apps show strong promise in increasing access to psychological support due to their availability, flexibility, scalability and relatively low price (14, 15). In particular, unguided mental health apps (also termed fully automated mental health apps), which can be used in the absence of a clinician, can potentially decrease clinicians' workloads (16).

There are various types of mental health apps available on the market. Self-guided, unguided, self-supported or fully automated apps are apps without human support and are entirely dependent on self-use. Guided apps are used with the support of a healthcare professional. Apps are sometimes used in the context of blended therapy, which is an approach that uses “elements of both face-to-face and Internet-based interventions, including both the integrated and the sequential use of both treatment formats” (17). In blended therapy, smartphone apps are used as only part of the treatment plan and aim to support and augment face-to-face therapy.

Theory-driven and evidence-based mental health smartphone apps show promising signs of efficacy in delivering digital therapy. One meta-analysis found that both guided and unguided smartphone interventions can reduce anxiety (18). Other studies revealed that digital health interventions, including smartphone apps, showed significant clinical improvements in depression and anxiety (19), and psychosis (20). Although research shows that apps with human support are more effective than automated digital interventions (21), guided apps could potentially increase healthcare professionals' workload (16). As such, there is a tradeoff between ensuring psychological support and the provision of therapy that is scalable and accessible, whilst balancing staff workloads and availability of face-to-face resources.

The therapeutic alliance (TA), also termed working alliance (22), refers to the relationship between a healthcare professional and a client, and is considered to be a fundamental factor in face-to-face psychological therapy. The most well-known conceptualization of TA is Bordin's theory (22), which suggests that TA is composed of three components: agreement on *goals, bonds* between healthcare professionals and clients, and agreement on the *tasks* that need to be undertaken to achieve goals. The Working Alliance Inventory (WAI), a scale that was developed based on Bordin's conceptual model, is commonly used to measure TA in face-to-face therapy. Another scale that has been used to measure TA is the Agnew Relationship Measure (ARM), which comprises five dimensions: bond, partnership, confidence in therapy, client initiative, and openness (23). TA is an important component of building engagement (24) and improving clinical outcomes in face-to-face therapy. Previous research has found that the TA generally has moderate but reliable correlations with clinical outcomes across all types of mental health problems and treatment approaches in both young people and adults (25–27).

Although researchers primarily understand TA as occurring in the context of face-to-face therapy, Bordin (22) argued that a TA can exist between a person seeking change and a change agent, which may not be a human healthcare professional. Interpreting this in a modern digital context, we propose that agents other than human healthcare professionals, including mental health smartphone apps, can possibly be such change agents for clients seeking change. However, the in-person healthcare professional is not present in fully automated mental health apps meaning that a potentially important mediator of change is not present. Therefore, understanding whether the concept of digital therapeutic alliance (DTA) exists in the fully automated mental health app context, and how it may differ from the TA in traditional face-to-face therapy, is important in understanding how fully automated apps can be developed to lead to better clinical outcomes.

Whether a TA can be formed in the digital context is unclear, and the concept of a DTA is nascent and under-researched. Theories from Human-Computer Interaction (HCI) which explain how individuals relate to digital technologies suggest the potential for DTA by explaining how apps can build empathy (28), persuade users to perform tasks and support them in task achievement (29), provide the flexibility that facilitates the availability of therapy (14), promote attachment to the app (30) and support self-determination (31). All of these theories may play a part in helping to understand how a DTA can evolve.

A few studies have investigated the TA in the digital context with different digital interventions, such as apps, internet-based CBT, and virtual reality (VR). Most quantitative studies have used the traditional in-person TA scales, such as the WAI (32) and Agnew Relationship Measure (14), to analyze the TA in the digital context, while some studies created DTA instruments by editing WAI or ARM (33–37). Several reviews have concluded that in a range of digital health interventions, the ratings of alliance are generally as high as in face-to-face therapy (25, 38–40). However, some studies have not reported meaningful correlations between ratings and clinical outcomes in the digital context (32, 36, 41–43). A handful of studies have attempted to measure the DTA with mental health smartphone apps, and we are aware of only one review that focused specifically on the DTA with both guided and fully automated smartphone apps (44). Henson et al. (44) found only five papers that met their inclusion criteria for review; only one of these studies measured the DTA with a scale/measure. More research has been conducted in this area since this review, and no published reviews have focused specifically on examining the DTA in the context of fully automated mental health smartphone apps. In addition, Henson and colleagues' review focused on serious mental illness. However, a broader range of mental health problems also needs to be considered.

Therefore, the objectives of this narrative review are to: (1) integrate the extant but growing literature on the DTA in the context of fully automated mental health smartphone apps; (2) examine the research gaps; (3) identify future research directions in the investigation of DTA as applied to fully automated mental health smartphone apps.

## Methods

A narrative review was conducted using PsycINFO, PubMed and Google Scholar to search for relevant literature. The databases were searched from inception to August 2021. DTA related articles were searched by using the snowball sampling and citation network analysis method (45–47). Lecy and Beatty (45) argued that in a given research topic, researchers should first find highly cited publications as seed articles. The seed articles should be highly cited and be several years old so that they can be exposed to a broad range of audiences. In the next stage, to expand the number of relevant papers, researchers find papers which cite the seed articles; and then in the second round, find the papers which cite the papers that cite the seed articles, and so on. They further suggested the process needs to be conducted over four rounds. While literature review can be limited by cognitive biases when using keywords, snowball sampling and citation network analysis offers a comprehensive approach to map a broader range of literature (45). Thus, to reach a broad range of studies, snowball sampling and citation network analysis was considered suitable for our study.

Specific Keywords (see Table 1) were used to search for seed articles. After identifying the seed articles, relevant papers that cited the seed articles were kept in the first round, then the relevant papers citing the first-round articles were kept in the second round, and so on. Four rounds of searching were conducted and all abstracts were examined to decide whether the full text needed to be further examined in detail. After four rounds, papers which were clearly within the topic scope at full text were selected for reviewing. Relevant articles were selected for inclusion in the review based on the following eligibility criteria. Inclusion criteria: (i) studies that investigated DTA related concepts in the context of fully automated mental health smartphone apps; (ii) both quantitative studies and qualitative studies. Exclusion criteria: (i) papers that only focused on text, email, online counseling, and video-conferencing; (ii) papers that only focused on guided apps or apps used in the context of blended therapy; (iii) studies that involve apps, but also require other digital devices, such as virtual reality (VR) headsets; (iv) reviews of papers.

**Table 1:** Search terms for the narrative review.

The following search phrases were used:
“therapeutic alliance” OR “working alliance” AND “digital”,
“therapeutic alliance” OR “working alliance” AND “mhealth”,
“therapeutic alliance” OR “working alliance” AND “computerized”,
“therapeutic alliance” OR “working alliance” AND “mobile”,
“therapeutic alliance” OR “working alliance” AND “technology”,
“therapeutic alliance” OR “working alliance” AND “smartphone”,
“therapeutic alliance” OR “working alliance” AND “internet”,
“therapeutic alliance” OR “working alliance” AND “app”,
“therapeutic alliance” OR “working alliance” AND “ehealth”,
“therapeutic alliance” OR “working alliance” AND “computer”,
“therapeutic alliance” OR “working alliance” AND “web”,
“therapeutic alliance” OR “working alliance” AND “automated”.

## Results

Twenty highly cited articles (minimum citation count of 100) on the topic of DTA (not only DTA in the context of fully automated mental health smartphone apps) were selected as seed articles. Those seed articles were highly cited and were published several years ago, so they have become central works on the topic of DTA. That said, all articles on the topic of DTA, including articles about DTA in the context of fully automated mental health apps, would cite those seed articles. In this way, we were able to reach a broad range of studies.

After four rounds of snowballing and citation network analysis, six studies were identified within the eligibility criteria. However, due to the under-researched nature of the topic of DTA in the context of fully automated mental health apps, none of the six identified articles had enough citations to become seed articles. Three of them were quantitative studies, two of the studies were qualitative studies, and the remaining study used a mixed methods approach. Basic study characteristics and conclusions are outlined in Table 2.

**Table 2:** Basic study characteristics and conclusions.

**Study**	**Study design**	**Intervention**	**Participants**	**DTA scale**	**Engagement measure**	**Conclusions**
Berry et al. (48)	Qualitative study	Actissist, a CBT informed app for people who have experienced a first episode of psychosis.	Stage 1: 9 Actissist users; Stage 2: 14 Actissist users and 10 mental health staff.	mARM	None	Developed mARM to measure DTA in the context of smartphone apps.
Goldberg et al. (49)	Study 1: cross sectional study and; Study 2: randomized controlled trail.	Study 1: Smartphone-based meditation apps in the market, such as Calm and Headspace. Study 2: Smartphone based Medication app—Healthy Minds Program (HMP).	Participants were in general population in both of the studies. *N =* 290 in study 1 and *N =* 314 in study 2.	DWAI	App Utilization. Study 1: self-report using frequency (daily, weekly, monthly, several times a year, or never); Study 2: Objective usage data gathered from the app.	DWAI correlates with frequency of app use (r = 0.42) in study 1, and correlates with HMP usage in study 2 (rs = 0.17–0.22). Early DWAI (week 1 and 2) didn't predict post treatment distress, but DWAI in weeks 3 and 4 associated with the clinical outcomes (βs= -.17 and -.13).
Clarke et al. (14)	Secondary Analysis of a Randomized Controlled Trial	Fully automated apps—myCompass	Participants were people with mild-to-moderate depression, anxiety, and/or stress symptoms. *N =* 90.	ARM	Number of program interactions (i.e., logins); number of modules completed; frequency of self-monitoring.	The scores of ARM did not correlate with clinical outcomes. TA subscales composite score was significantly positively correlated with engagement (r = 0.32–0.38).
Prochaska et al. (50)	Randomized controlled trial	CBT based Chatbot app (Woebot) with tracking and notification functions.	Participants were 8–65 years old and screened positive in substance misuse (scoring>1 on the CAGE-AID). *N =* 180	WAI short form revised	Usage data metrics: days used, in-app text messages, and completed modules.	Greater frequency of use (total numbers of in-app text) was weakly associated with a reduction in substance use occasions (r = 0.23).
Darcy et al. (51)	Cross-sectional, Retrospective Observational Study	CBT based Chatbot app (Woebot) with tracking and notification functions.	Participants were Woebot users in general population. *N =* 36,070	WAI short form revised	None	The mean of bond sub-score is 3.84 which is comparable to face-to-face therapy. Thus, there is a possibility that users can build bond with apps.
Hillier (52)	Qualitative study	All types of unguided technology based interventions, including fully automated apps.	Participants were people with variety of clinical issues, including depression, anxiety, and bipolar disorder. *N =* 13	None	None	Participants generally rejects the ideas of having bonds or relationships with technology based interventions.

### Ways in Which DTA Has Previously Been Measured in the Context of Fully Automated Mental Health Apps

Two studies measured the DTA using the WAI short form and one study measured DTA using the ARM. In addition, two measurements, the Mobile Agnew Relationship Measure (mARM) (48) and the Digital Working Alliance Inventory (DWAI) (49) have been proposed to measure the DTA in fully automated mental health apps. The mARM was created by replacing the word “therapist” with “app,” and adding, deleting, and rewording some questions based on qualitative feedback from both clients and healthcare professionals (48). The DWAI is a short 6-item survey based on the WAI, and was created by choosing two items from each subscale of WAI and replacing the term “therapist” with “app” (53).

### Relationships Between DTA and Clinical Outcomes

Only two identified studies examined the relationships between the DTA and clinical outcomes in the context of fully automated mental health apps, with mixed findings. Goldberg et al. (49) assessed the DTA by using the DWAI in fully automated meditation apps and found that DWAI scores at weeks 3 or 4 only correlated with reductions in psychological distress (βs = −0.17 and −0.13). However, Clarke et al. (14) examined the relationship between the DTA (measured by the ARM) and clinical outcomes with a fully automated mental health app, comprising educational modules and multiple other functions, and found no statistically significant correlation. These mixed findings are inconsistent with findings from face-to-face therapy (25–27). One possible explanation is that the DTA is somewhat different from the TA, and the existing DTA scales are not comprehensively measuring all aspects of the DTA. Even if some studies found correlations between the ratings of the scales and clinical outcomes, this could be by chance. For example, Goldberg et al. (49) used the six-item DWAI to measure the DTA with meditation apps. However, the DWAI was seemingly developed by choosing two items from each subscale of WAI short form without employing a formal scale construction method. Thus, whether the DWAI comprehensively/adequately measures DTA is questionable.

### Potential Differences in Characteristics Between TA and DTA in the Context of Fully Automated Mental Health Apps

First, bond is considered a critical element in the face-to-face TA conceptual model and is a subscale of both the WAI and ARM. However, the role of bond in the DTA is unknown. One qualitative study found that users generally rejected the ideas of having a connection or a bond with fully automated digital mental health interventions (52). In a study of the fully automated app myCompass, Clark et al. (14) found that the ratings of the bond subscale measured post-treatment did not predict clinical outcomes, while the task and goal subscales moderately correlated with clinical outcomes. However, two studies showed that users could potentially build a bond with chatbot apps. A study of the app Woebot (a therapeutic chatbot that delivers CBT), which was used to reduce substance misuse during the COVID-19, found that the bond subscale rating was higher than task and goal subscales ratings (50). Similarly, another study with Woebot found that the bond subscale rating was high and comparable to face-to-face therapy (51).

Second, some factors of the in-person TA are difficult to achieve in the digital context. Two studies which used qualitative interview methods found that although apps can mimic human support in some ways, users felt that apps were less understanding (14, 52).

On the other hand, some positive feelings, such as the sense of not being judged and the feeling of being accompanied, can be more easily derived from DTAs. Three studies which used qualitative research methods found that users felt more comfortable interacting with technology than healthcare professionals because participants were less fearful about being judged and consequently felt less stigmatized (14, 48, 52). In addition, by interviewing users, two studies found that interactivity might be an important component of DTA which was not a TA subscale (14, 52). Clark et al. (14) also argued that flexibility in time, location and duration, is a characteristic that apps can provide beyond human health professionals.

### Impact of DTA on Engagement

TA is fundamental in building adherence or engagement with therapy (24, 54, 55). However, the importance of adherence and engagement with mental health apps remains unclear and there is no standard way of measuring engagement with mental health apps. Two studies defined engagement as app usage. However, the types of usage data in these two studies were different. Clarke et al. (14) measured number of program interactions (i.e., logins), number of modules completed, and frequency of self-monitoring, while Prochaska et al. (50) measured days used, in-app text messages, and completed modules. In addition, Goldberg et al. (49) adopted self-report usage frequency as engagement in study 1, and adopted usage data in study 2. They did not further explain what types of usage data were gathered.

Only one study analyzed correlations between engagement and outcomes. A study of the chatbot app Woebot, used to help reduce substance misuse, found that greater frequency of use (numbers of in-app text) was not significantly associated with a reduction in substance use occasions (r = 0.23) (50).

To the best of our knowledge, two studies have measured the correlations of engagement and DTA in fully automated apps and they used different measurement approaches. Clarke et al. (14) found that, in the fully automated myCompass app, the TA (measured by ARM) subscales composite score was significantly positively correlated with engagement, when engagement was measured by logins, numbers of modules completed and frequency in self-monitoring (r = 0.32–0.38). Goldberg et al. (49) found that in fully automated meditation apps, DWAI scores significantly positively related to app utilization, which was measured by either self-reported user frequency (r = 0.42) or usage data gathered from the app (rs = 0.17–0.22).

## Discussion

TA originally refers to the relationship that can develop between healthcare professionals and clients; the concept has been mostly used in face-to-face therapy. Researchers suggest that a form of TA may exist in the digital context (48), but a further explication of the nature and quality of alliance is needed. Some researchers have examined the DTA in the context of fully automated apps by using or slightly modifying face-to-face therapy measurement scales, such as the ARM and WAI. However, the nature of DTA is unclear. Although two studies showed that DTA was positively associated with engagement, the approaches for measuring engagement were inconsistent. In addition, previous studies did not show consistent and reliable correlations between DTA and clinical outcomes in the context of fully automated apps (14, 49). This finding is at odds with the conclusions drawn from face-to-face therapy where there is a robust association between alliance and outcomes. One possible explanation is that the DTA is somewhat different from the TA, and the existing DTA scales are not comprehensively measuring all aspects of the DTA. Even if some studies found correlations between the ratings of the scales and clinical outcomes, this could be by chance.

It is worth noting that an app can lead to positive clinical outcomes without building a DTA. However, it is still possible that the app can bring better or more reliable clinical outcomes when it can build a DTA with users. In addition, some researchers have argued that a positive correlation between TA and clinical outcome at one time point was not sufficient in proving the importance of TA in face-to-face therapy (56). Moreover, apps outcomes might not be direct clinical outcomes, but rather indirect outcomes via promotion of behavior changes, such as increasing help seeking behaviors. Thus, the association between DTA and clinical outcomes, and how to assess the importance of DTA, requires further investigation. However, the priority is re-conceptualizing the DTA in the context of fully automated mental health apps.

Current evidence suggests that the conceptualization of a DTA in the context of fully automated apps in the first place may differ in significant ways from that of the traditional TA for the following reasons: (1) the role of bond in the DTA is unknown; (2) some human components of face-to-face TA, such as empathy, are hard to achieve in the digital context; and (3) some users may perceive apps as more non-judgmental and flexible, which may further influence DTA formation. For example, researchers found that time convenience and interactivity can enhance users' relationship commitment with smartphone apps (57). Considering the above discrepancies, it appears that differences exist between DTA and in-person TA. Thus, a new scale exploring the nuances of the DTA in the context of fully automated apps is warranted.

Herrero et al. (35) suggested that modifying the words in TA scales is not sufficient to develop new DTA scales. According to Boateng et al. (58), the first step of developing a new scale should be defining the domains of the scale. Therefore, instead of modifying TA scales, researchers need to understand what are the key components of a DTA. Multiple questions need to be answered. For example, is it possible for users to build a bond or an attachment with an app? Can users and apps agree on goals? What are the components that apps can provide beyond therapists? The subdisciplines of computer science and HCI theories mentioned in the introduction may help us to answer some of these questions.

### Affective Computing: Building an Empathic App

While empathy is fundamental to building client-therapist TA, clinicians have expressed concerns around building empathy in the context of fully automated digital health interventions (14). Affective computing, defined as “computing that relates to, arises from, or influences emotions” (59), could possibly help to build DTA in the following three ways.

First, affective computing allows computers to detect users' emotions through text, which can further help computers understand users' individual needs. Bradley and Lang (60) created the Affective Norms for English Words (ANEW), which provided emotional and affective ratings to a large number of English words. Similarly, computer scientists developed a WordNet-Affect to identify whether a word is positive or negative (61). Emotion can also be detected in physiological ways. Calvo and Peters (62) indicated that emotion can be detected from measures of heart rate, respiration, blood-pressure, skin conductance, and so on. One of the benefits of smartphones and wearable devices is that they contain sensors, which can help to detect and track users' physiological states and detect emotions states. This allows apps and accompanying wearable devices to tailor activities or wellness suggestions to users in response to their changing emotional state (63) in a way that is similar to providing customized plans by human healthcare professionals.

Second, according to narrative empathy theory (28), digital health interventions are able to share empathy by using appropriate design and wordings. Narrative empathy is “the sharing of feeling and perspective-taking induced by reading, viewing, hearing, or imagining narratives of another's situation and condition” (28). Designers can express their empathy by using high-impact graphs (62) and creating scenarios (64). For example, Wright and McCarthy (64) argued that novels and films can usually draw people's empathic feelings, so in technology, designers can use novel-like scenarios to share empathy. Choosing text appropriately is another method by which designers show empathy and enhance the user experience. In a study of an app designed for Syrian refugees, the users expressed their fondness for the language with a Syrian accent, since it provided users the feelings of interacting with real people (65). Similarly, users of the app myCompass expressed their favor of the empathic voice of the content (14).

Third, relational agents (RAs), which can mimic human healthcare professionals' behavior, could be built based on affective computing. Relational agents are computer programs that can have conversations and potentially build relationships with users (66). An embodied agent aims to “produce an intelligent agent that is at least capable of certain social behaviors and which can draw upon its visual representation to reinforce the belief that it is a social entity” (67). A non-embodied agent is a text-based agent (68), and sometimes also named a chatbot. Bickmore and Gruber (66) further argued that RAs can build long-term relationships with clients when using certain design strategies, such as variability and self-disclosure. A review of mobile health interventions concluded that people automatically respond to computers in social ways and relational agents can be used to develop TA by providing empathy and respect (69). In a study of a health education and behavior change counseling app, researchers found that users felt the embodied conversational agents can mimic human, and can further lead to better DTA (70). Some researchers found that users can trust, have the feeling of being cared for, and build an alliance with various types of relational agents based tablet/computer apps, such as alcohol and substance misuse counseling apps, and stress management apps (71–74). Similarly, Suganuman et al. (75) used an embodied relational agent in a fully automated mental health app and found it was effective in bringing positive outcomes. They further argued that the RAs can enhance the DTA.

Computer scientists have also developed relational agents based on the theory of rapport. Rapport is believed to be a relationship quality that occurs during the interactions in crowds. It can be built via both verbal (prosody, words uttered, etc.) and non-verbal behaviors (nodding, directed gaze, gesture, etc.) (76). Researchers further found that relational agents, which were built based on the theory of rapport, can induce users' openness (77, 78). As openness is a subscale of ARM, relational agents can potentially help to enhance DTA by building the rapport with users.

However, users may also not trust relational agents. Concerns, such as cybersecurity and information accuracy, stop some people from using and trusting the relational agents (79). Thus, how to further utilize relational agents to build DTA needs to be further explored.

### Persuasive Technology

#### Building Task and Goal With Persuasive Technology

Goals and tasks are subscales of WAI, and could also be dimensions of DTA, since some studies found that ratings of task and goal link with clinical outcomes in the context of fully automated mental health apps (80, 81). Persuasive technology can be used to enhance tasks and goals in the context of fully-automated mental health apps.

Persuasive technology, also termed behavior change technology, was defined as “any interactive computing system designed to change people's attitudes and behaviors” (82). Fogg (82) further argued that computers can be (1) tools that change users' attitudes and behaviors by making desired results easier to achieve; (2) media which can provide stimulated experiences to change users' behavior and attitudes; and (3) persuasive social actors which can trigger social responses in humans. In addition to the roles of computers, Fogg (82) further stressed the importance of mobility and connectivity, and believed that persuasiveness can be increased by interacting with the right people/things at the right time.

Drawing on Fogg's work, Oinas-Kukkonen and Harjumaa (29) introduced a Persuasive Design System Model, which is divided into four categories:

- Primary task support: This category is employed to support users to achieve their primary tasks. It includes reduction, tunneling, tailoring, suggestion, self-monitoring, surveillance, conditioning and rehearsal.
- Dialogue support: Dialogue support is employed to provide feedback to users and includes praise, rewards, reminders, suggestions, similarity, liking, and social role.
- System credibility support: This category described the ways of designing a computer or system to make it more credible, and consists of trustworthiness, expertise, surface credibility, real-world feel, authority, third-party endorsement, and verifiability.
- Social support: This category builds upon Fogg's mobility and connectivity theory and contains social learning, social comparison, normative influence, social facilitation, cooperation, competition, recognition.

Primary task support strategy is used to make task completion easier. Theoretically, this strategy can be used to help users to complete tasks and achieve their goals, which would further strengthen the DTA. In addition, in face-to-face therapy, one important strategy of building positive TA is customized treatment plans for different individuals (83). In a similar way, tailoring and personalization in primary task would enhance DTA. Previous research also supports this idea. Researchers found that personalization and tailoring features were highly requested by users (84, 85). Self-monitoring in primary task support strategies is favored by users. Clinicians found that the mood-tracking feature of BlueWatch, a mobile app for adults with depressive symptoms, received positive reviews from users (86). Moreover, self-monitoring has also proved effective. For example, a study using smartphone apps for treating eating disorder symptoms found a standard self-monitoring app led to significant improvements in outcomes (87).

Kelders and other researchers (88) found that the more dialogue support used the better the adherence. Reminders and notifications particularly contributed to higher adherence. A meta-analysis found that the apps with reminders have significantly lower attrition rates (89). From the DTA perspective, reminders and notifications can help users focus on completing tasks and achieving their goals. For example, one study of an app for reducing alcohol consumption found that the feedback function was highly rated by clients (87).

#### Building Flexibility With Persuasive Technology

Flexibility (in terms of using time, location, duration, the way of using and interacting with apps) is a characteristic that apps can provide beyond human health professionals (14). Clarke et al. further (14) suggested that flexibility should be added as a subscale when conceptualizing the DTA in the future. Persuasive technology strategies can help fully automated apps maximize their flexibility and potentially further enhance DTA. One of the advantages of having flexibility is that users can get support whenever and wherever they want. Dialogue support strategy can be applied to the apps in order to provide real-time responses. Tailoring and personalization in primary task support strategy can also help apps to provide better flexibility in the way of allowing users to choose their own way of using apps. For example, some users liked the design of the myCompass app which allowed users freely chose structured programs or self-paced study (14).

### Human—App Attachment: Building Bond or Attachment With Mental Health Apps

As a subscale of both WAI and ARM, *bond* is considered critical in face-to-face therapy. However, whether and how users can build a bond with apps remains unclear as some users rejected the idea that they could form a relationship with digital mental health interventions (52). Thus, understanding how to help users build an attachment or a bond would be important in forming DTA in the context of fully automated apps.

Li et al. (90) indicated that when clients suffer from pain, illness, tiredness, and anxiety, they tend to seek external help and may bond with health applications. Some features, in particular, are believed to help build human-app attachment. Computer scientists have argued that users can trust, be open to, and keep a long-term relationship with relational agents (77, 78, 91, 92). In a study of technology for older people, participants described their experiences with relational agents as talking to a friend (30). Zhang and other researchers (93) found that through interactions with the device and personalized feedback, users can form an emotional bond with mobile health services. Game elements and gamification may be another solution for creating human-app attachment, as studies reported that users can build an attachment with customized game characters (94, 95).

Persuasive technology can also be applied to help users build bonds with apps. For example, a study of a smartphone app designed for children with Autism Spectrum Disorders found that user-app attachment could be enhanced by persuasive technology techniques (96). A system credibility support strategy in persuasive technology is also needed in building a bond. Trust and respect are considered important in building deeper bonds with healthcare professionals, which will further enhance client-therapist therapeutic relationships (22, 97). Thus, a system credibility support strategy can strengthen DTA by helping users build trust and respect in relation to mental health apps. In a qualitative study of a blended therapy app for men with intentional self-harm, Mackie et al. (98) found that trust in the function and effectiveness of the applications is crucial for building TA with apps rather than with people. Additionally, some items in the mARM are relevant to this strategy. For instance, the question – *I have confidence in the app and the things it suggests* requires trustworthiness, authority, and expertise principles.

However, not all users can build an attachment or a bond with digital health interventions (52). Kim et al. (95) argued that human—app attachment is influenced by the self-connection and social-connection that users can get through apps. Users have a greater possibility to build an attachment with apps when they feel the apps express who they are and offer them close relationships with the social world. The attachment style of individuals may also influence human-app attachment. Attachment style originally describes how people think, react, and behave in relationships with other people (99). In recent years, researchers also found that attachment style can be applied in the context of artificial intelligence (AI) and information technology (IT) (100, 101). For example, Gillath et al. (100) pointed out that people with attachment anxiety can have less trust in AI. However, how these theories can help users to build attachment with fully automated mental health apps requires further investigation.

### Positive Computing and Positive Psychology

Positive psychology studies show how positive human functioning, such as life-purpose, self-realization and self-knowledge can contribute to well-being (102). They show out that well-being or happiness can be measured subjectively. Self-determination theory (SDT) in positive psychology examines how inherent human capabilities and psychological needs are influenced by biological, social, and cultural conditions (103). Although SDT contains multiple mini theories, the most well-accepted and well-known one is the Basic Psychological Needs Theory. Ryan and Deci (31) believe people can be motivated by the satisfaction of competence, relatedness, and autonomy. Competence refers to the feeling of effectiveness in interaction; relatedness refers to feeling connected with and being cared for by other people; autonomy refers to being the source of one's own behavior or value (31).

Positive computing is a computing area concerned with studying how to develop technology to support human well-being (62). It usually incorporates eudaimonic or positive psychological theories, such as SDT, into the process of designing technology (104). Motivation and TA are believed interrelated in face-to-face therapy (105, 106). Thus, positive computing that draws on SDT may contribute to DTA formation in fully automated mental health apps. Goldberg et al. (49) argued that in fully automated mental health apps, motivation boosting content can be given to users to enhance DTA when users report a low alliance rating. Lederman et al. (107) promoted the idea, finding that SDT-based online platform design can provide support for TA. SDT can also be interlinked with persuasive technology to enhance DTA. Villalobos-Zúñiga and Cherubini (108) argued that persuasive technology features link with SDT. They categorized persuasive technology features based on SDT:

- Autonomy: reminders, goal setting, motivational messages, pre-commitment
- Competence: activity feedback, history, log/self-monitoring, rewards
- Relatedness: performance sharing, messaging

In summary, multiple areas of computer science and HCI provide various theories and methodologies to build DTA in fully automated mental health apps, and developers and scientists should choose appropriate approaches depending on the determinant factors and purpose of the apps.

### Limitations

This review has some limitations. First, only six identified studies examined DTA on fully automated mental health apps. Thus, it is still unclear whether the conclusions reached in these studies can apply to all types of fully automated mental health apps. In addition, since this is not a systematic review, there is a possibility that not all relevant literature has been captured. However, in examining and drawing out the similarities between these various related contexts we have raised some valuable points for consideration. Second, we did not include studies that examined DTA in tablet/computer apps. We acknowledge that tablet/computers apps can be similar to smartphone apps. However, their differences are significant in terms of our investigation. For example, tablets/computers are not as flexible/convenient as smartphones. In addition, tablets/computers may not be as accessible as smartphones. There is a larger population owning smartphones than owning tablets. These factors can influence the ways that users build DTA with apps. Thus, there is a risk that DTA in the context of fully automated smartphone apps may differ from tablets/computers. Third, we acknowledge that historically, there have been many critiques around the concept of TA. Some researchers have questioned the importance of TA in therapy, and have criticized Bordin's conceptual model (56, 109, 110). However, the TA is still a well-established concept, and has been measured in many studies (including studies of digital health interventions). Thus, our arguments in this paper were all based on the assumption that TA is a central factor in therapy.

### Future Directions

In terms of future directions, many questions need to be answered to understand the development of DTA with fully automated mental health apps, but the priority is in formulating a valid conceptualization of DTA and how to formally measure it. A new qualitative interview-based study focused on fully automated mental health apps users is needed to re-conceptualize and redefine the DTA. Once a new comprehensive scale of the DTA has been developed, which can be administered in the context of fully automated mental health apps, the impact of DTA can be further investigated, including whether the DTA predicts outcomes and engagement. Choosing accurate approaches of measuring engagement is necessary when examining the relationship between DTA and engagement. Moreover, whether engagement is important to lead to better clinical outcomes needs to be further studied. Melvin et al. (111) indicated that an app for suicide prevention is still effective to help users cope with suicidal thoughts even if the users had only used it on one occasion. In addition, selecting appropriate methods to assess the association between DTA and clinical outcomes is also important. Furthermore, testing the DTA on various types of fully automated mental health apps is also essential. Whether the concept of DTA is influenced by different therapy methods also needs to be further investigated. For example, do the ways of building DTA differ in the interventions informed by CBT and the apps informed by dialectical behavior therapy (DBT)? Studies investigating what app features can contribute to DTA formation could also be carried out in the future.

## Conclusion

In conclusion, understanding DTA is critical in examining how fully automated mental health smartphone apps can be developed to lead to better clinical outcomes. Currently, this topic is under-researched and more studies are needed. We found that the conceptualization of DTA may differ from TA for three reasons. First, the role of bond in the context of fully automated apps is unclear. Second, human components of face-to-face TA, such as empathy, are hard to achieve in the digital context. Third, some users may perceive apps as more non-judgmental and flexible, which may further influence DTA formation. Thus, the priority for future research is to develop a new DTA scale which can be administered in the context of fully automated mental health apps. Subdisciplines of computer science, such as affective computing and positive computing, and some human-computer interaction theories, such as those of persuasive technology and human-app attachment can potentially help researchers to understand DTA formation in fully automated apps.

## Author Contributions

FT performed the article selection, developed the theoretical formalism, and took the lead in writing the manuscript. RL, SD'A, KB, and SB equally contributed to the research by providing critical feedback, helping shape the research, and editing the manuscript. All authors contributed to the article and approved the submitted version.

## Funding

FT was supported by a University of Melbourne and University of Manchester Graduate Research Group PhD Scholarship.

## Conflict of Interest

SB is a director and shareholder of CareLoop Health Ltd, which develops and markets digital therapeutics for mental health problems. The remaining authors declare that the research was conducted in the absence of any commercial or financial relationships that could be construed as a potential conflict of interest.

## Publisher's Note

All claims expressed in this article are solely those of the authors and do not necessarily represent those of their affiliated organizations, or those of the publisher, the editors and the reviewers. Any product that may be evaluated in this article, or claim that may be made by its manufacturer, is not guaranteed or endorsed by the publisher.
